# Bilateral sternobronchial fistula after coronary surgery – are the retained epicardial pacing wires responsible? a case report

**DOI:** 10.1186/1749-8090-4-26

**Published:** 2009-06-24

**Authors:** Timothy Sakellaridis, Michalis Argiriou, Victor Panagiotakopoulos, Christos Charitos

**Affiliations:** 1 2nd Cardiac Surgery Department, "Evagelismos" General Hospital, Athens, Greece

## Abstract

**Background:**

Temporary epicardial pacing wires are routinely used during cardiac surgery; they are dependable in controlling postoperative arrhythmias and are associated with low morbitity.

**Case report:**

We report a case of sternobronchial fistula formation induced by the existence of retained epicardial pacing wires in a patient who underwent coronary surgery ten years ago.

**Conclusion:**

Reported complications of retained epicardial pacing wires are unusual. We present this case in order to include it to the potential complications of the epicardial pacing wires.

## Background

Sternal wound infections account among the most serious complications after median sternotomy for cardiac operations. Late studies published, report tremendous morbidity with an incidence of 0.4 to 5% [1,2] and in-hospital mortality between 7–29%, even when mediastinitis was correctly treated [3].

The use of temporary epicardial pacing wires during cardiac surgery is a routine procedure and has been associated with low morbitity [4]. We describe a rare case of a fistula formation with communication from the substernal space to the skin and to the left and right bronchial tree. We believe the retained epicardial pacing wires being responsible.

## Case report

A 70-year-old Caucasian male, with a medical history of lung emphysema and occlusion of the left anterior descending coronary artery (LAD), underwent in 1997 an off-pump coronary artery bypass grafting (OPCAB) procedure with the left internal mammary artery anastomosed to the LAD. Atrial and ventricular temporary epicardial pacing wires were placed as accustomed. The patient's postoperative course was uneventful. Before discharge, the pacing wires were cut after gentle traction at the skin level and allowed to retract.

One year later the patient presented with an inflamed type V wound according to the El Oakley & Wright classification [2], at the lower part of the sternotomy. He underwent operation with removal of two sternal wires, and surgical debridement. Closed circuit irrigation with antimicrobial and antibiotic solutions was applied and antibiotics were systematically administered. The patient was discharged ten days later with no sign of inflammation.

**Figure 1:**
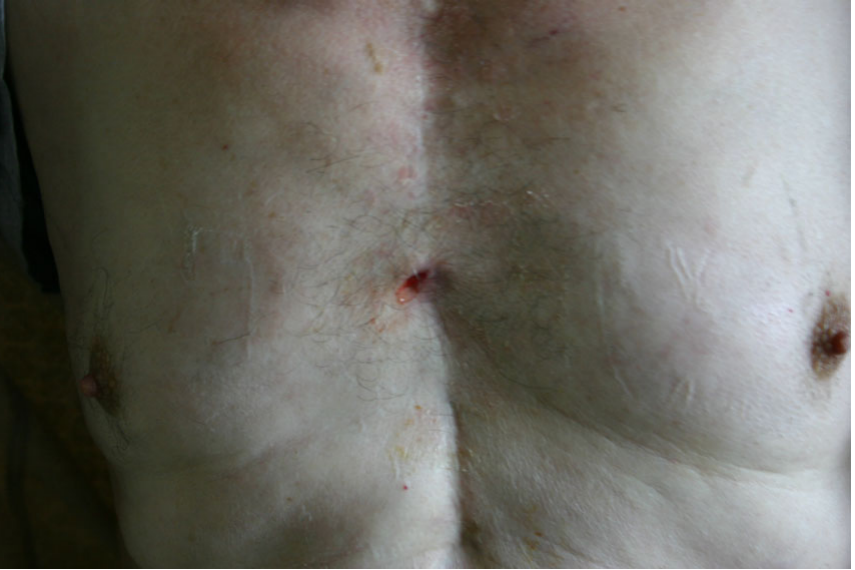
**The existence of a small free draining pustule along the lower part of the sternotomy**.

**Figure 2:**
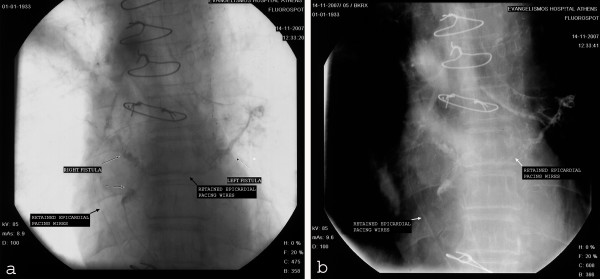
**Fistulography of the cutaneous sternal fistula showing filling of a small substernal cavity with contrast medium and communication with both bronchial trees and the presence of the retained epicardial pacing wires along the fistula**.

Nine years after he proceeds complaining for cough and for a small free draining pustule along the lower part of the sternotomy (Figure 1). The chest radiograph and computed tomography (CT) were diagnosed as normal. The fistulography revealed a cutaneous sternal fistula filling a small substernal cavity with contrast medium and communication with both bronchial trees. The presences of the retained epicardial pacing wires along the fistula are characteristic. The sternobronchial fistulas were with the superior division of the lingular bronchus at the left lung and at the right with the bronchus of the upper lobe (Figure 2). The general condition of the patient was good with no fever, no pneumonia, no sepsis and no pain. Cultures of the pustules identified the presence of Staphylococcus aureus. The patient received antibiotic therapy with resolvement of the free draining pustules.

Surgical removal of the retained epicardial pacing wires and debridement was recommended for treatment. Due to the resolution of the free draining pustules with the administration of the antibiotics, the age of the patient, the lung disease (emphysema with FEV_1 _= 1,2 l) and the patient's refusal to any surgical interventions, surgery was not performed. Eight months after he has no signs of sternal wound infection and is in good condition.

## Conclusion

Temporary pacing wires are routinely sutured to the atrial and/or ventricular myocardium during open heart surgery. These wires are of value for both diagnosis and treatment of cardiac arrhythmias following surgery. Their use may facilitate improved cardiac output by maintenance of an appropriate cardiac rate or atrio-ventricular sequence. This practice is based upon the assumption that the risks are low in comparison with the benefits. The complications of epicardial pacing wires use are with an occurrence rate of 0.4% [4]. Reported complications of the pertinent literature include failure to rhythm sense or capture, myocardial bleeding of pacemaker wire site introduction site, avulsion of a haemostatic clip from the saphenous vein graft, perforation of the superior epigastric artery, right ventricular laceration [4], infection, migration of a wire into the pelvis [5], laceration of the saphenous vein graft [6], cardiac decompensation during removal of a wire ensnaring the heart [7], migration of a retained wire in a bronchus [8] and in a lung [9] and transepidermal migration [10].

The incidence of sternal wound infection after cardiac surgery is reported to be 0.4–5% [2]. Sternal wound infections are classified according to the guidelines of the Center for Disease Control and Prevention (CDC), and deep sternal wound infections are classified according to the criteria proposed by El Oakley and Wright [2].

The incidence of a sternobronchial fistula, a fistula communicating from the substernal space to the skin and to the bronchial tree is an extremely rare complication after cardiac procedures. Searching the pertinent literature we were able to find only one other case [11]. In our case, the retained epicardial pacing wires in collaboration with the sternal wound infection presented one year after the surgery and the history of lung emphysema are responsible for the formation of the fistula.

The use of temporary epicardial wires are a commonplace in cardiac surgery. Care is taken that the wire ends are cut clean without fraying and that there is an adequate redundant loop in the pericardial cavity that allows cardiac movement for distal anastomoses inspection or reopening the chest. We ensure that no saphenous vein graft lies in proximity to any bare wire, especially a right coronary bypass graft with hemoclips on side branches. Care is taken in the placement of mediastinal and pericardial chest tubes so that the temporary wires are not looped proximal to the chest tubes and they are placed near the midline to avoid the superior epigastric artery. As for the removal, it takes place one day prior discharge with gentle traction of the atrial and ventricular pacing wires. If there is a resistance we cut them at the skin level and allowed the wires to retract.

In case of mediastinitis or deep sternal wound infection, total excision of the sternum including all corresponding costal cartilages, lung resection and resection of the chest wall and using of muscle flaps would be the recommended, as a radical surgical treatment, if the patient's condition can tolerate it. A reserved disposition must be taken and the possibility of lung resection in such cases is reserved only for extreme cases with direct parenchymal dissemination or pleural empyema. Another recommended procedure would be, surgical removal of the retained epicardial pacing wires and debridement of the sternal wound. In all cases administration of the proper antibiotics after culture of the pustules is mandatory and the surgical correction depends on the wound and the patient's condition.

We present this case in order to include the formation of fistulas as a potential complication of epicardial pacing wire use.

## Consent

Written informed consent was obtained from the patient for publication of this case report and any accompanying images. A copy of the written consent is available for review by the Editor-in-Chief of this journal.

## Competing interests

The authors declare that they have no competing interests.

## Authors' contributions

TS, MS, VP & CC designed the study protocol, carried out the clinical assessment, and drafted the manuscript. All authors read and approved the final manuscript.
